# Total face mask with neurally adjusted ventilatory assist as a rescue therapy in infants with severe bronchiolitis

**DOI:** 10.1007/s00431-024-05543-1

**Published:** 2024-04-06

**Authors:** Vladimir L. Cousin, Tiphaine Corbisier, Peter C. Rimensberger, Angelo Polito, Alice Bordessoule

**Affiliations:** Paediatric and Neonatal Intensive Care Unit, Department of Paediatrics, Gynecology and Obstetrics, University Hospital of Geneva, University of Geneva, Rue Gabrielle-Perret-Gentil 4, 1206 Geneva, Switzerland

**Keywords:** NAVA, Non-invasive ventilation, Bronchiolitis, Full-face mask, PICU

## Abstract

Severe bronchiolitis patients are often supported with non-invasive ventilation (NIV). In case of NIV failure, we recently started to use non-invasive neurally adjusted ventilatory assist ventilation (NIV-NAVA) with a total face mask interface (TFM) and report now our experience with this modality of respiratory support. Retrospective study was made from October 2022 to May 2023 at the Geneva University Hospital Paediatric Intensive Care Unit. Inclusion criteria were children, aged from 0 to 6 months, with severe bronchiolitis with initial NIV failure and switch to NIV-NAVA-TFM. From 49 children with respiratory syncytial virus (RSV)-induced bronchiolitis requiring any form of respiratory support, 10 (median age 61 days (IQR 44–73) failing CPAP or NIV underwent rescue treatment with NIV-NAVA using a TFM. Patients were switched to TFM-NIV-NAVA 8 h (IQR 3–22) after admission for 24.5 h (IQR 13–60). After initiation of TFM-NIV-NAVA, oxygenation improved significantly as early as 1 h after initiation, whereas transcutaneous CO_2_ values remained stable. None of the patients needed to be intubated and there was no episode of TFM discontinuation due to interface discomfort or other unwanted side effects. Sedation was used in all patients with high proportion of intravenous dexmedetomidine. Median ventilatory assistance duration was 2.5 days (IQR 2–4) and median PICU stay was 4.5 (IQR 3–6).

*  Conclusion*: In infants with severe RSV-induced bronchiolitis, respiratory support with TFM-NIV-NAVA seems to be feasible as a rescue therapy and might be considered in selected patients.

**Table:** 

**What is Known:** *• Bronchiolitic patients with NIV support failure may require invasive mechanical ventilation.* *• Interface related complications, especially facial sores, can be a cause of NIV failure.*	
**What is New:** *• Total face mask with non-invasive neurally adjusted ventilatory assist (TFM-NIV-NAVA) seems feasible as a rescue therapy in deteriorating patients with CPAP or NIV failure.* *• TFM-NIV-NAVA can improve oxygenation rapidly in patients with aggravating hypoxemia and seems to be well tolerated.*

**Supplementary Information:**

The online version contains supplementary material available at 10.1007/s00431-024-05543-1.

## Introduction

Bronchiolitis is a leading cause of respiratory failure in infants and non-invasive ventilation (NIV), including continuous positive airway pressure (CPAP), is one of the recommended respiratory supports [1]. However, some patients require invasive mechanical ventilation [2]. Patient-ventilator asynchrony and intolerance of the interface could emerge as a significant issue during NIV and lead to NIV failure. Non-invasive neurally adjusted ventilatory assist (NIV-NAVA) has demonstrated its effectiveness in enhancing patient-ventilator interactions and lessening the effort required for breathing [3, 4]. Nasal prongs or nasal masks are the most used NIV interfaces, although alternative options like total face masks (TFM) are available [5, 6]. TFM cover the mouth, the nose, and the eyes and have been successfully used in adult with acute respiratory failure, with enhanced comfort and reduced air-leaks [7, 8]. Smaller TFM are available and their use in the bronchiolitis has shown to be promising [5].

Our local practice is to use NIV-NAVA with TFM-interface in case of CPAP or NIV therapy failure at attending physician’s discretion to optimize both interface and respiratory support. The aim of our study was to describe our experience with the use of TFM in conjunction with NIV-NAVA for infants with severe bronchiolitis unresponsive to initial NIV support.

## Methods

### Population

This is a retrospective study of infants under 6 months of age, admitted in pediatric intensive care unit (PICU) for a severe bronchiolitis failing initial NIV support. The study period spanned from October 2022 to June 2023, maintaining a consistent team of nurses and physicians during this period. The study followed Helsinki Declaration standards for human experimentation and the local ethic committee (Commission Cantonale d’Ethique de la recherche, CCER number 2023-00230, March 20th, 2023) approved the study and waived the need for consent.

### Interventions

Using non-vented nasal mask (supplementary materials), CPAP or NIV settings were adjusted at the discretion of the attending physician, except positive end expiratory pressure (PEEP) levels, which were set at 7 cmH_2_O [9]. In case of CPAP or NIV failure, patients were switched to TFM-NIV-NAVA or intubated as determined by the attending physician. CPAP or NIV failure was defined as increasing respiratory distress, worsening hypoxemia (FiO2 > 0.4), or significant discomfort persisting despite appropriate sedation. Upon initiation of total face mask ventilation, feeds are discontinued due to the potential need for intubation within the subsequent hours. Following a period of stability, typically exceeding 12 h, feeds are gradually reintroduced.

A TFM mask (PerforMax^®^, Respironics Inc., Murrysville, PA, USA) was chosen as interface as it might reduce air leaks and improve patient comfort while covering the full face. If not already in place, a NAVA naso-gastric probe (Maquet Critical Care, Solna, Sweden) was inserted. Initial NIV-NAVA settings were as follows: inspiratory trigger 0.5 µV and a default expiratory trigger at 70% of the peak electrical activity of diaphragm (EAdi). Maximal initial pressure limit was set at 25 cmH_2_O. NAVA support level and FiO_2_ were set according to the attending physician preference, while PEEP level is maintained at 7 cmH_2_O [9].

For ventilation interface tolerance, hydrocolloid dressings are routinely used to protect patients’ skin, both in case of nasal mask and TFM. Their use is tailored to patient’s individual anatomy. Oral or intravenous sedation was prescribed as needed. In addition, non-pharmaceutical strategies are used to improve patients’ comfort (supplementary materials).

Respiratory rate (RR), oxygen saturation/inspired oxygen ratio (SpO_2_/FiO_2_) [10], transcutaneous CO_2_ (TcCO_2_), and NIV-NAVA levels of support were analyzed at time of TFM-NIV-NAVA initiation and after 1, 2, 4, 6, 12, and 24 h. For each variable, the average of data gathered during the most recent 15 min within every hour was used, as they were collected by a computerized ICU system (Centricity Critical Care Clinisoft^®^, GE Healthcare, USA).

### Statistical analysis

Continuous and categorical variables are expressed as median and interquartile range (IQR) and proportion (%) respectively. Wilcoxon paired rank test was used for group comparison. Prism GraphPad v. 9.0 (GraphPad software, San Diego, CA, USA) was used for statistical analysis.

## Results

**Fig. 1:**
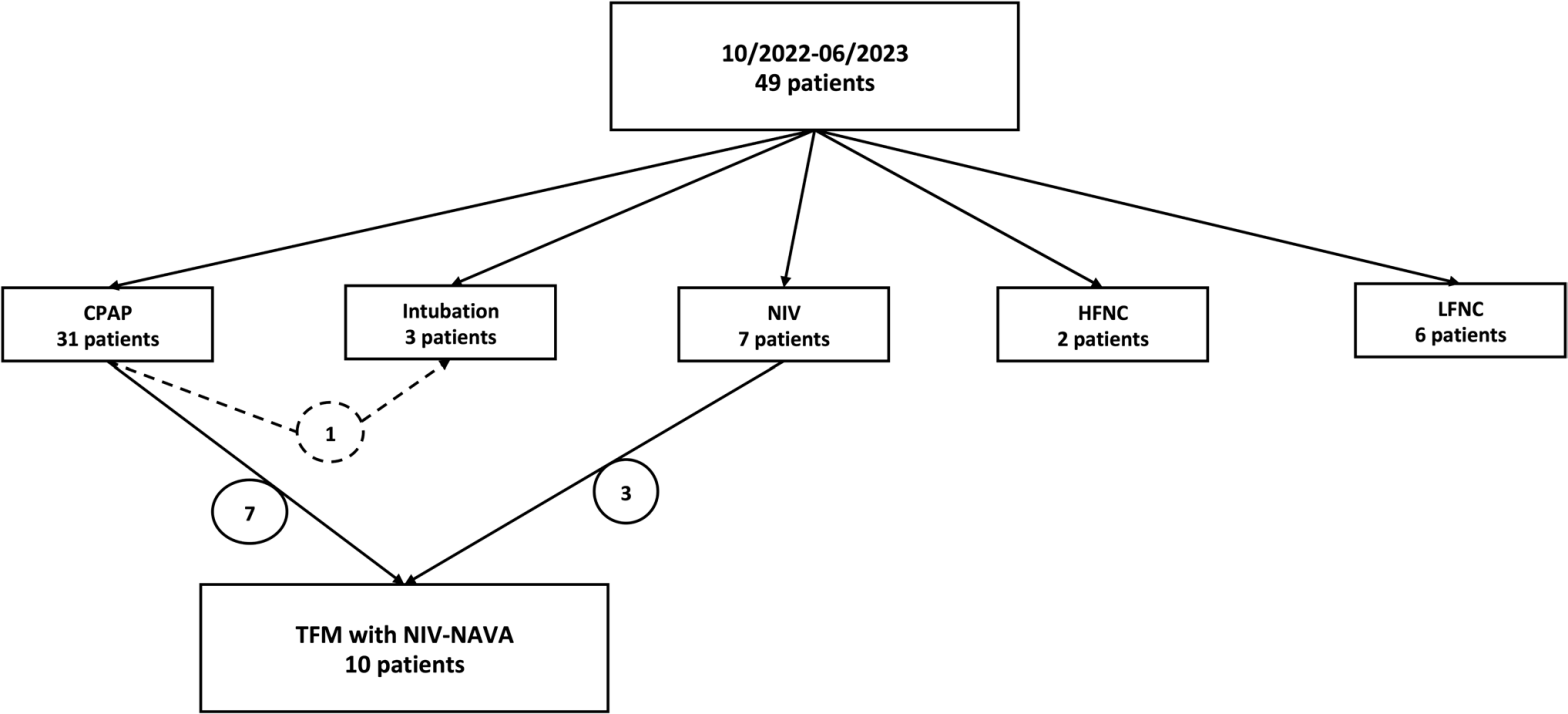
Study flowchart, with the initial respiratory support 1 patient from CPAP was intubated. 7 patients from CPAP group and 3 from NIV group were switch to TFM-NIV-NAVA. CPAP continuous positive airway pressure; HFNC high flow nasal cannula; LFNC low flow nasal cannula; NAVA neurally adjusted ventilatory assist; NIV non-invasive ventilation; TFM total face mask

Over the study period, 49 patients were admitted in PICU. Of those, 10 patients underwent clinical deterioration while on CPAP and/or NIV, which prompted the transition to TFM-NIV-NAVA (Fig. 1). At admission, median age was 61 days (IQR 44–73), median capillary pH was 7.29 (IQR 7.24–7.32), and median capillary pCO_2_ was 7.9 kPa (IQR 7.2–8.8).

Median time from admission to TFM-NIV-NAVA was 8 h (IQR 3–22). Indications to switch to TFM-NIV-NAVA were increased respiratory distress (8/10), worsening hypoxia (3/10), and nasal mask intolerance (2/10). Patients could cumulate more than 1 criteria. The median length of TFM-NIV-NAVA support was 24.5 h (IQR 13–60). None of these patients required intubation.

Sedation was administered in 8/10 patients at baseline, with 3/8 receiving it orally and 5/8 intravenously. At 2 h of follow-up (H2), all patients required sedatives. When used, dexmedetomidine dose increased over time (Supplementary Fig. 1-A).

**Fig. 2:**
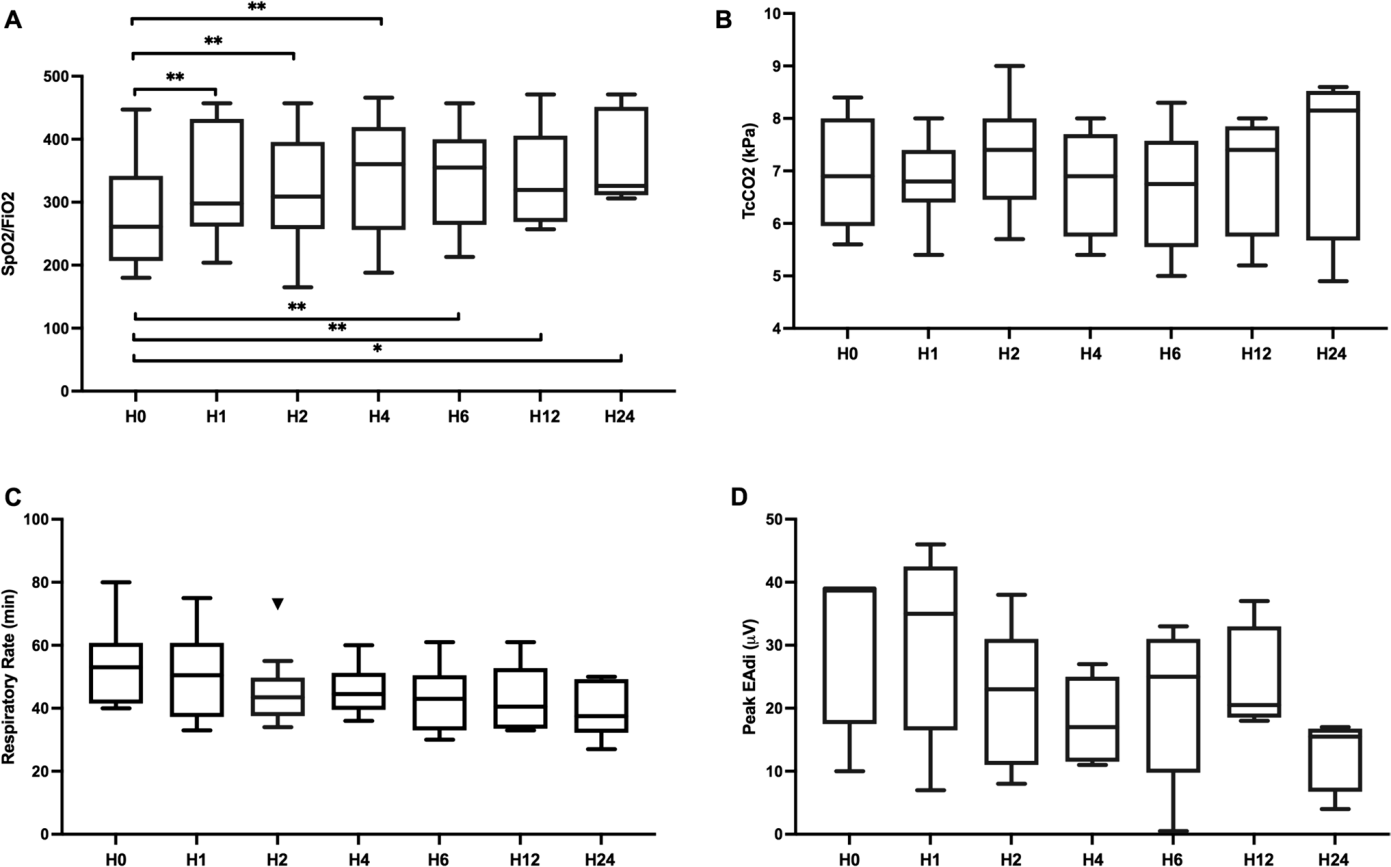
Evolution of SpO_2_/FiO_2_, TcCO_2_, respiratory rate, and peak EAdi under TFM-NIV-NAVA support. Time point were H0 at initiation of TFM, then after 1 h (H1), 2 h (H2), 4 h (H4), 6 h (H6), 12 h (H12), and 24 h (H24). Boxplot depicted using Turkey method. Panel **A** SpO2/FiO2 evolution. Panel **B** TcCO2 evolution. Panel **C** respiratory rate evolution. Panel **D** Peak EAdi. *P* value * < 0.05 ** < 0.01

The evolution of SpO_2_/FiO_2_, TcCO_2_, and RR is depicted in Fig. 2A–C. TFM-NIV-NAVA allowed for a significant oxygenation improvement seen as early as 1 h after commencing TFM-NIV-NAVA support. During this time, TcCO_2_ and RR remained steady. Initially, median NAVA level was 0.6 (IQR 0.5–0.8) and median initial peak EAdi value was 39 µV (IQR 25–39). Peak EAdi, PIP, and NAVA level, depicted in Fig. 2D and supplementary Fig. 1B–C, remained stable over time.

Following TFM-NIV-NAVA, patients underwent transitions to various modes of respiratory support: nasal CPAP 4/10 nasal NIV-NAVA and nasal NIV-PS 3/10 for each. Overall, total ventilation duration of was 2.5 days (IQR 2–4), including CPAP support time, and PICU length of stay was 4.5 days (IQR 3–6).

## Discussion

In this single-center report, we observed that TFM with NIV-NAVA might be a viable option for patients with severe bronchiolitis unresponsive to initial CPAP and/or NIV support. Moreover, TFM-NIV-NAVA use showed improvement in oxygenation.

Our findings do not provide sufficient evidence to ascertain whether the observed improvement in oxygenation can be attributed to NIV-NAVA, through reduction of patient-ventilator asynchrony [3], to the TFM that might minimize air-leaks, or the combined effect of both interventions. Considering NIV-NAVA as a viable optimization of NIV support over conventional NIV in patients with bronchiolitis has been suggested by others. It improved patient-ventilator synchrony and limit inefficient effort, both factors being linked to NIV failure [3, 4]. Adding a TFM interface might offer additional advantages over traditional nasal or facial masks [5]. Observed improved oxygenation might result from a more efficient transmission of applied airway pressure due to reduced air leaks with TFM coupled with a more effective delivery of the intended FiO_2_. All these factors might help to reduce intubation rates [3–5, 8].

We did not report any facial sores in our cohort, a frequent complication with the use of more traditional interfaces [11]. Facial sores can sometimes limit the pursuit of NIV and TFM have been described used with good tolerance and comfort despite extensive facial sores in adults [7]. Hence, utilizing TFM may offer an efficient way for managing facial sores and the continuation of NIV support while circumventing the need for intubation. Interface intolerance, often cited as a common reason for NIV failure, emerged as the main cause of failure in the TRAMONTANE study [11]. For this reason, sedatives are frequently used in children with bronchiolitis as it was the case in our population also. Moreover, sedation could play a specific role in this population as it may limit patient-ventilator asynchrony and reduce the pathologic respiratory drive, reducing the occurrence of self-inflicted lung injury [12].

Several limitations to the present study must be mentioned, including its retrospective nature and the small number of patients. Criteria to switch on TFM-NIV-NAVA were not fully standardized. Improvement of oxygenation could be partly due to progressive spontaneous recovery, although this latter seems not to be very likely as a rapid improvement after switching to TFM-NIV-NAVA was observed in all patients. Finally, the applicability of our findings could be limited due to the need for a specialized nurse team, along with a low nurse-patient ratio.

## Conclusion

Our results suggest that using TFM-in conjunction with NIV-NAVA might be favorable and might be considered in a rescue attempt in CPAP or NIV failure. This combination seems to be feasible even in critically ill infants and has the potential to improve oxygenation in severe bronchiolitis. Our findings warrant additional research to substantiate the advantages of this approach in terms of minimizing the requirement for intubation and invasive ventilation.

### Supplementary Information

Below is the link to the electronic supplementary material.

Supplementary file1 (DOCX 327 KB)

## Data Availability

Data may be available from the corresponding author after approval by the senior author on reasonable request.
