# Supplementary material for: Total face mask with neurally adjusted ventilatory assist as a rescue therapy in infants with severe bronchiolitis
Source: Eur J Pediatr. 2024 Apr 6;183(7):2813–7. doi: 10.1007/s00431-024-05543-1 (PMC11192665; doi:10.1007/s00431-024-05543-1)
Supplement: Supplementary file 1 — Supplementary file1 (DOCX 327 KB) [file 431_2024_5543_MOESM1_ESM.docx]

**Supplementary Materials**

**Page 2: supplementary methods materials**

**Page 3: supplementary figure 1**

**Supplementary methods materials**

**Respiratory support**: initial mask was a non-vented nasal mask (Respireo Soft® nasal mask, Air Liquide Healthcare, Air Liquide Medical Systems, Antony, Fr) and active humidification (Infant Ventilator Circuit RT 266, Fisher & Paykel Healthcare, Auckland, NZ). Attending physician could choose between different respiratory support: high flow nasal cannulas at 2 liter/kg/min, continuous positive airway pressure (CPAP), non-invasive ventilation (NIV) with a positive end-expiratory pressure (PEEP) and a peak pressure (NIV-PS) and NIV-Neurally adjusted ventilatory assist (NAVA). As part of a unit protocol, all PEEP levels are set at 7 cmH_2_O.

**Ventilators**: Two types of ventilators were used: Maquet Servo-I® and Maquet Servo-U® (Maquet critical care, Solna, Sweden), both before and during TFM-NAVA-NIV support. Vital signs (respiratory rate and heart rate) were continuously monitored. SpO_2_ (Oximax NN; Coviden, Elancourt, France) and transcutaneous pCO_2_ (TcPCO_2_) (Tina TCM 4/40; Radiometer Medical ApS, Brønshøj, Denmark) were also measured.

**Sedation**: If needed, we use a protocol of oral and intravenous sedation, set by the attending physician. Paracetamol is routinely used as a first line analgesic and anti-fever treatment (15 mg/kg/6h hours). In addition, hydroxyzine (1 mg/kg/8 hours) and/or clonidine (1 mcg/kg/8 hours) were used for oral sedation, and dexmedetomidine for intravenous sedation (0.3-1 mcg/kg/h). The degree of sedation was adapted by the attending nurse and physician, using mostly Comfort B score.

Apart from pharmaceutical interventions, we implemented various environmental strategies to enhance patients' comfort. Whenever feasible, patients were accommodated in single rooms, ideally with a nurse-to-patient ratio of 1, ensuring personalized care and attention. To minimize auditory disturbances, we utilized noise-activated warning signs, aiming to maintain a tranquil environment conducive to healing. Additionally, we regulated lighting levels, dimming them when active patient care was not underway, thereby fostering a calm and soothing atmosphere.

**Supplementary figure 1.**

**Evolution dexmedetomidine dose, peak inspiratory pressure and NAVA level under TFM-NIV-NAVA support**


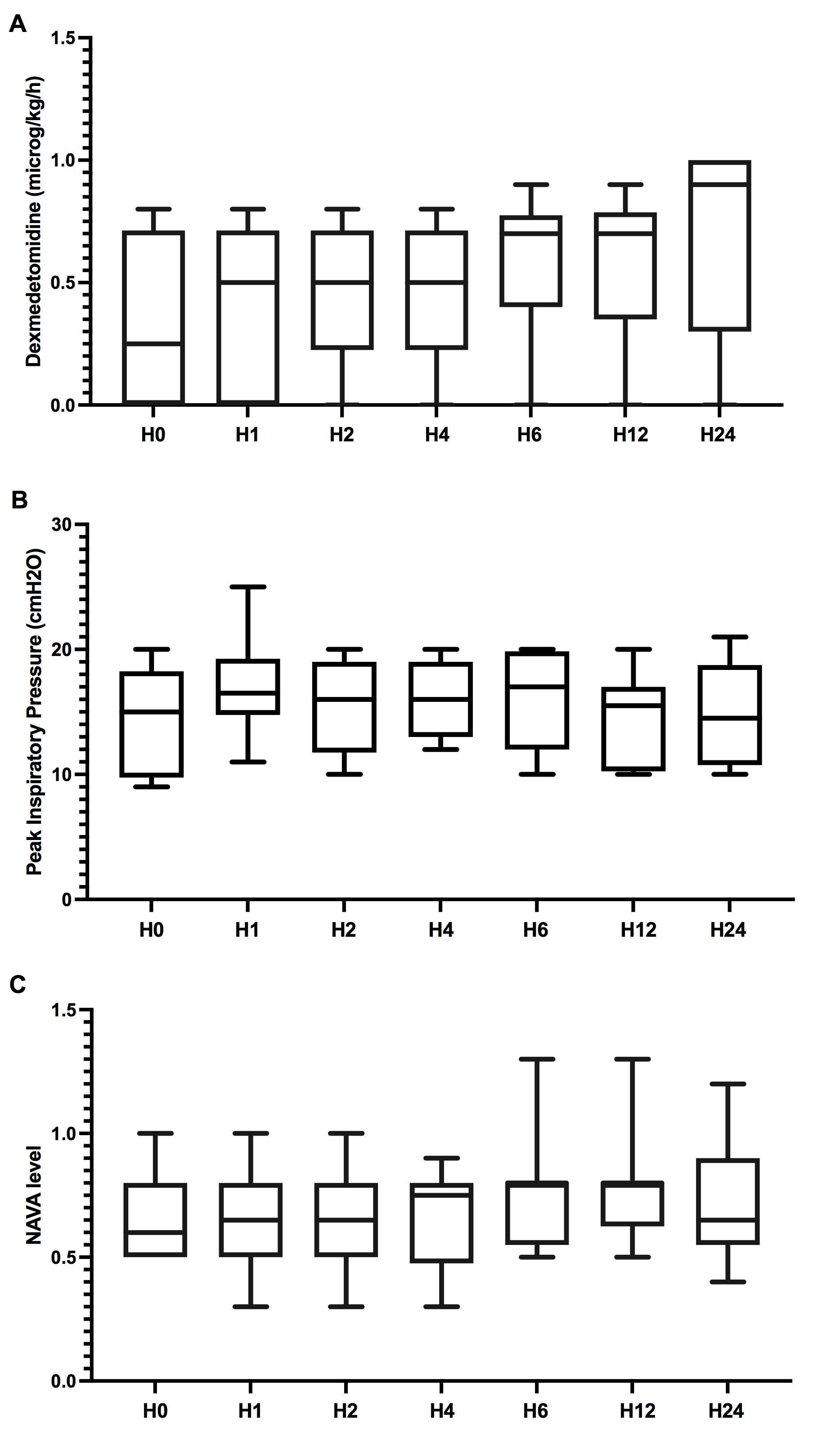


Panel A Dexmedetomidine dose evolution, Panel B Peak inspiratory pressure evolution, Panel C NAVA level evolution. Boxplot depicted using Turkey method.

Time point were H0 at initiation of TFM, then after 1 hour (H1), 2 hours (H2), 4 hours (H4), 6 hours (H6) 12 hours 8H12) and 24 hours (H24).
